# Can CD44 Be a Mediator of Cell Destruction? The Challenge of Type 1 Diabetes

**DOI:** 10.1371/journal.pone.0143589

**Published:** 2015-12-01

**Authors:** Nathalie Assayag-Asherie, Dror Sever, Marika Bogdani, Pamela Johnson, Talya Weiss, Ariel Ginzberg, Sharon Perles, Lola Weiss, Lora Eshkar Sebban, Eva A. Turley, Elimelech Okon, Itamar Raz, David Naor

**Affiliations:** 1 The Lautenberg Center for Immunology and Cancer Research, IMRIC, The Hebrew University-Hadassah Medical School, Jerusalem 91120, Israel; 2 Department of Endocrinology, Hadassah University Hospital, Ein Kerem, Jerusalem 91120, Israel; 3 Matrix Biology Program, Benaroya Research Institute, Seattle, WA, United States of America; 4 Department of Bone Marrow Transplantation and Cancer Immunotherapy, Hadassah University Hospital Ein Kerem, Hebrew University, Jerusalem, 91120 Israel; 5 London Regional Cancer Center, University of Western Ontario, London, ON, Canada; 6 LEM laboratories Science Park, Rehovot, Israel; 7 Diabetes Unit, Hadassah University Hospital, PO Box 12000, Jerusalem 91120, Israel; University of British Columbia, CANADA

## Abstract

CD44 is a multi-functional receptor with multiple of isoforms engaged in modulation of cell trafficking and transmission of apoptotic signals. We have previously shown that injection of anti-CD44 antibody into NOD mice induced resistance to type 1 diabetes (T1D). In this communication we describe our efforts to understand the mechanism underlying this effect. We found that CD44-deficient NOD mice develop stronger resistance to T1D than wild-type littermates. This effect is not explained by the involvement of CD44 in cell migration, because CD44-deficient inflammatory cells surprisingly had greater invasive potential than the corresponding wild type cells, probably owing to molecular redundancy. We have previously reported and we show here again that CD44 expression and hyaluronic acid (HA, the principal ligand for CD44) accumulation are detected in pancreatic islets of diabetic NOD mice, but not of non-diabetic DBA/1 mice. Expression of CD44 on insulin-secreting β cells renders them susceptible to the autoimmune attack, and is associated with a diminution in β-cells function (e.g., less insulin production and/or insulin secretion) and possibly also with an enhanced apoptosis rate. The diabetes-supportive effect of CD44 expression on β cells was assessed by the TUNEL assay and further strengthened by functional assays exhibiting increased nitric oxide release, reduced insulin secretion after glucose stimulation and decreased insulin content in β cells. All these parameters could not be detected in CD44-deficient islets. We further suggest that HA-binding to CD44-expressing β cells is implicated in β-cell demise. Altogether, these data agree with the concept that CD44 is a receptor capable of modulating cell fate. This finding is important for other pathologies (e.g., cancer, neurodegenerative diseases) in which CD44 and HA appear to be implicated.

## Introduction

Type 1 diabetes (T1D) is in many aspects a representative inflammatory autoimmune disease, which can be used as a model for unveiling the mechanism of action underlying chronic inflammation in general. The etiology of T1D is to some extent a puzzling topic. However, most if not all investigators agree that this disease displays clear adaptive and innate autoimmune parameters, leading to destruction of the insulin-secreting β cells, e.g., by reactive oxygen species (ROS), such as nitric oxide (NO)[1,2,3,4,5,6].However, the intra-islet invasion mechanisms by pre-diabetic inflammatory cells and the process underlying the demise of β cells undergoing attack by the immune system have yet to be understood. A clearer picture of these pathological activities could provide useful insight not only for T1D but also other chronic inflammations and pave the way for new therapies.

The concept of leukocyte transendothelial migration has been previously described [7,8,9,10,11] and the role of chemoattractants [4] and integrins [12] in T1D has been well demonstrated. Yet, less is known about the role of CD44 in this pathology [13].

Alternative splicing generates multiple functions and structures (isoforms) of CD44, including standard CD44 (CD44s), which is the shortest and ubiquitous version of this glycoprotein [14,15]. We previously reported [16] that a structurally different protein-receptor, hyaluronic acid mediated motility (RHAMM; CD168), compensates for CD44 when CD44 is genetically deleted in the collagen-induced arthritis model. RHAMM is expressed both on the cell surface and inside the cell, where it regulates intracellular signaling of cell motility, cell division and microtubule stability. RHAMM, like CD44, binds HA and supports cell trafficking [17,18].

Injection of anti-CD44 monoclonal antibody (mAb) or hyaluronidase reduced the diabetic activity in young male NOD recipients reconstituted with inflammatory female spleen cells [13]. Further, the findings suggest that the interaction between cell surface CD44 of the inflammatory cells and hyaluronic acid (HA) of the tissue is an essential step in the process of cell invasion into the pancreatic islets and development of T1D.

Here we describe a yet unreported observation: the severity of T1D in NOD mice is dependent on a balance shifted in favor of CD44 expression on insulin-secreting β cells, which induces β cell apoptotic destruction under inflammatory conditions. The universal implications of our findings are discussed.

## Materials and Methods

### Mice

NOD/Ltj mice and CD44-deficient C57BL/6 mice [19] were purchased from Jackson Laboratories (Bar Harbor, ME, USA). In our facility, female NOD mice become diabetic at a median age of 16–17 weeks, with 95% diabetic by 30 weeks of age. In order to examine the role of the CD44 molecule in the pathogenesis of T1D, CD44-deficient NOD mice were generated by back-crossing the CD44 deletion from CD44-deficient C57BL/6 mice onto the NOD background for 12 generations. To assure homogeneity of the NOD background and rule out the potential effects of C57BL/6 genetic contamination, microsatellite and single nucleotide polymorphism (SNP) analyses of the CD44-deficient NOD mice genome were combined, according to the MGI database (http://www.informatics.jax.org). To this end, the CD44 gene, located on chromosome 2 at base pair position 54.13 cM, was flanked by SNPs at position 20.0, 31.0, 32.0, 33.0, 35.0, 38.0, 39.0, 40.0, 41.0, 42.9, 45.9, 47.3, 51.1, 53.08, 54.0, 55.1, 55.9, 56.9, 58.1, 59.05, 63.3 and 69.8 cM. All those SNPs were homozygous for NOD markers. In addition, the other chromosomes were screened for the following microsatellite markers: D1Mit18, D1Mit130, D3Nds6, D3Mit95, D6Mit31, D6Mit135, D6Mit198, D7Mit20, D7Mit328, D8Mit205, D9Mit25, D10Mit87, D11Mit339, D11Mit298, D13Mit16, D13Mit61, D14Mit110, D14Mit222, D17Mit175, D17Mit34, D18Mit171, D18Mit202, D18Mit208, D18Mit4 (http://www.informatics.jax.org). All those microsatellites were found homozygous for NOD markers. In all the experiments, wild type littermates-CD44-positive mice- were used as reference mice. NOD females, 8 to 10 weeks-old, were defined as pre-diabetic mice, although they exhibited insulitis at this age. CD44-deficient and WT NOD males were used as recipient mice in the cell transfer experiments (see below). CD44-deficient DBA/1 mice were generated by backcrossing CD44-deficient C57BL/6 mice into DBA/1 mice (Harlan, Jerusalem, Israel) for 8 generations [16]. All animals were maintained in a specific pathogen free research animal facility, and the experiments were conducted in accordance with local ethical guidelines of the Hebrew University Institutional Animal Care and Use Committee. The Hebrew University is an AAALAC International accredited institute. The protocol was approved by Hebrew University Institutional Animal Care and Use Committee (IACUC), permit number MD-12-13535-4.

### PCR analysis

Tail-tip DNA from WT and CD44-deficient NOD mice was analyzed by PCR (MJ research Inc., Waltham, USA). The samples were amplified for 35 cycles of 1 min denaturating at 94°C, 30 sec annealing at 62°C, 1 min 30 sec extension at 72°C and a final extension of 10 min. WT (GGC CAA CTT CAT TTG GTC CAT GGT) and common (TTG AAT GTA ACC TGC CGC TAC GCA) primers produce a 110-bp band for CD44 and common and neomycin (TAT CAG GAC ATA GCG TTG GCT ACC) primers produce a 300-bp band in CD44-deficient mice. The PCR product(s) were analyzed by 1% agarose gel electrophoresis and Syber Safe^®^.

### Monitoring of diabetes

Diabetes was assessed by glycosuria determinations (> 1000 mg/dL in two consecutive measurements) (Teststrip from Medi-Test, Combi 9, Macherey-Nagel, Düren, Germany) or by blood glucose measurements, using a portable Ascencia Elite glucometer (Bayer, Basel, Switzerland). To measure blood glucose, we scored the tip of the tail (<2 mm incision) with a sterilized scalpel blade accordingly to the ethical guidance of the Hebrew University of Jerusalem. Additional measurements were performed by removing the clot from the first incision. Values above 14 mM (> 250 mg/dL) glucose in two consecutive measurements were considered as diabetic values. Spontaneous diabetes was monitored once a week for 30 weeks. Recipient mice included in adoptive transfer experiments were assessed for diabetes two to three times per week for 80 to 200 days.

### Adoptive transfer experiments

Cell transfer experiments were performed by by i.v. injection of 20x106 splenocytes from diabetic NOD females into young (6 to 8 weeks old) irradiated (550 rad, 5.5 cGy) NOD males that barely develop diabetes, unless reconstituted by the female cells (detailed description in [13]).

### Histopathology and cell infiltration scoring analysis

Pancreases from five NOD females in each group were fixed in 10% buffered formalin, embedded in paraffin and then, 5-μm sections (200 μm apart) were stained with hematoxylin and eosin. At least 17 islets or more were detected in each section and scored by an uninformed observer according to the following criteria: 0, no cell infiltration; 1, periinsulitis and cell infiltration involving up to 20% of islet area; 2, cell-infiltration involving up to 50% of islet area; 3, cell infiltration involving up to 75% of islet area; and 4, cell infiltration involving 90–100% of islet area. We have calculated the percent of islets showing the different scores by dividing the number of islets with score 1–4 by the number of islets analyzed in this individual mouse. Surprisingly, we found identical total number of islets in the WT and KO mice (754). Data was analyzed with Excel.

### Immunohistochemistry

Paraffin-embedded mouse pancreatic tissue, 5-μm sections, was deparaffinized and rehydrated. Heat-Induced Epitope Retrieval (HIER) was performed in EDTA solution pH 8 (Zymed Laboratories, Invitrogen Corporation, Carlsbad, CA,) in a pressure cooker (Decloacking ChamberTM, Biocare Medical, Concord, CA). Endogenous peroxidase was quenched in 3% solution of hydrogen peroxide (Perdrogen, Riedel-de Haën, Sigma-Aldrich, Slelze, Germany). Sections were incubated overnight with 5 μg/ml rat anti-CD44 mAb (eBioscience, San Diego, CA) or 4D2 (rat IgG2b) isotype-matched control mAb [16] in CasBlock solution (Zymed) at 4°C in a humidified chamber. Then the sections were incubated with N-Histofine Simple Stain Mouse MAX PO (Nichirei Biosciences Inc., Tokyo, Japan), 30 mins at room temperature. CD44 expression was detected with the DAB Plus Substrate System (Thermo SCIENTIFIC, Kalamazoo, MI). Sections were counterstained with hematoxylin and eosin before mounting. For HABP staining, pancreatic islet sections were incubated overnight at 4°C with biotinylated HABP (2.5 μg/ml; Calbiochem, LaJolla, CA). Then, the sections were incubated for 1 hr at room temperature with HRP-conjugated streptavidin (from Jackson ImmunoResearch, West Grove, PA). To assess the specificity of the staining with bHABP, slides were incubated 3 hrs at 37°C with testicular hyaluronidase (100 U/ml; Sigma, St. Louis, MO) prior to incubation with bHABP.

For the CD44 / Insulin double immunohistochemistry, sections were pre-treated for 15 minutes in citrate buffer pH6 in a pressure cooker. Sections were rinsed in PBS and were incubated in 2% Normal Donkey serum, 1%BSA in PBS for 30 minutes to block the nonspecific binding. Sections were then incubated overnight with antibodies to insulin (ab6995, Abcam) and CD44 (Thermo Scientific, #MA1-10225), used at dilutions 0.4μg/ml and 2μg/ml, respectively. The secondary antibodies Alexa Fluor 488 or Alexa Fluor 555 conjugated IgG (Molecular Probes, Grand Island, NY) were used at 3 μg/ml; the peroxidase-alkaline phosphatase-labelled IgG were used at 1:200. The WARP Red and deep space black chromogen kits (Biocare medical) were used to detect insulin and CD44, respectively. The nuclei were visualized with DAPI or by counterstaining with haematoxylin (Sigma, St. Louis, MO). Positive and negative controls were included in each staining experiment. Tissues were examined using a Leica DMIRB microscope and images were acquired using a Spot Xplorer^™^ camera and imaging software. All the images were taken under the same experimental settings and light exposure.

### TUNEL assay

Ten-week-old wild type and CD44-deficient normoglycemic NOD females were sacrificed and pancreases were harvested, flattened and immersed in 4% paraformaldehyde in ice for 4 hours, and then in cold ethanol 80% overnight. Tissues were embedded in paraffin the next day. Sections of 5 μm were rehydrated, and antigen retrieval was performed in citrate buffer pH = 6 in a pressure cooker. β cells were stained with guinea pig anti insulin (Abcam; 1/200) and apoptosis was assessed using the terminal deoxynucleotidyl transferase-mediated dUTP-biotin 3’ nick end-labeling (TUNEL) assay (Roche) according to the manufacturer instructions. Nuclei were stained with DAPI (1 μg/ml). Affinity-purified secondary antibodies were from Jackson ImmunoResearch. Immunofluorescence images were captured on an Olympus FlouView FV1000 confocal microscope at 400x magnification. Images of sections were analyzed using the ImageJ software.

### Isolation and culture of pancreatic islets

Mice (7-week-old) were killed by cervical dislocation immediately before islet isolation. The pancreata were perfused with 2–3 ml of collagenase XI (0.5 mg/ml; Sigma). Bile ducts were clamped off at the duodenal insertion to allow cannulation and specific perfusion into the pancreas. Perfused pancreata were digested for a batch-specific optimized time and collagenase activity was stopped with ice-cold HBSS (Biological Industries Inc., Beth Haemek, Israel). Islets were collected by handpicking under a dissecting microscope into RPMI 1640 complete medium, (containing 10%; heat-inactivated FBS GIBCO, Carlsbad, CA), 2mM L-glutamine, 100 U/ml penicillin, 100 μg/ml streptomycin, 10 mM HEPES, 1 mM sodium pyruvate and non-essential amino acids (Biological Industries).

### Transwell migration assay

Transwell migration assays in 5 μm-Costar plates (Corning Incorporated, Corning, NY) were performed as described [16]. 4x10^5^ cells were allowed to migrate overnight toward the bottom compartment containing a general chemoattractant (i.e., conditioned media derived from 293T cell line), to permit migration of all cell types. Cells that traversed the HA layer and dropped into the low chamber compartment were counted with FACS (FACScalibur, Becton Dickinson, Mansfield, MA). The number of cells that crossed the filters in the absence of chemoattractant was subtracted from chemo-attracted cells. In order to compare between different experiments, we normalized the data to the migration of the KO cells, where 100% refers to (the number of CD44-deficient cells that crossed the filter in the presence of chemoattractant—the number of CD44-deficient cells that crossed the filter in the absence of chemoattractant) x100.

### Exposure of pancreatic islet to pro-inflammatory cytokines and HA and assay for nitric oxide (NO) quantitation

Isolated islets were allowed to recover at 37°C overnight in complete RPMI medium. Then, they were incubated for different time periods (as indicated in the figure) in complete RPMI containing the pro-inflammatory cytokines cytokines IL-1β (50 U/ml), IFN-γ (1000 U/ml) and TNF-α (1000 U/ml) (R&D Systems, Minneapolis, MN) or medium. In a separate experiment (S3C Fig), the islet cells were pre-incubated overnight in serum-free medium supplemented with ITS+3 (Sigma) and containing rooster comb HA (300μg/ml, Sigma). Release of NO2- (a stable product of the reaction of NO. with molecular oxygen) from the cytokine- treated cells into culture supernatants was assessed by Griess assay as previously described [20].

### Glucose-stimulated insulin secretion

Glucose-stimulated insulin secretion (GSIS) was performed as previously described [21]. Briefly, for insulin secretion experiments, isolated islets cultured overnight in media, were washed with Krebs-Ringer bicarbonate HEPES (10 mM) buffer, 0.1% BSA and incubated for 2 h prior to glucose challenge. Four to 7 samples of 10 islets each were first exposed to Krebs buffer containing low glucose (3.3 mM) for 1 hour and then to high glucose (16.7 mM) for an additional 1 hour. To determine insulin content, islets were lysed in 500 μl ice-cold ethanol 75% + HCl 0.18N overnight. Insulin concentration in supernatant were determined using an Ultra Sensitive Mouse Insulin ELISA (Crystal Chem. Inc., Downers Grove, IL) performed according to manufacturer instructions.

### Western Blot

Isolated splenocytes or islets were lysed in protein sample buffer (15% glycerol, 60 mM Tris-HCl pH 6.8, 3% SDS, 7.5% ß-mercaptoethanol). Protein lysates were separated on 10, 12.5 or 15% SDS-PAGE and transferred to a Protran nitrocellulose membrane (Whatman Ltd., Dassel, Germany) or to Immobilon PVDF membrane (Millipore Corporation, Billerica, MA). After blocking in 5% nonfat dry milk, the membranes were incubated with the appropriate antibodies, namely anti-tubulin (Sigma), anti-CD44 (eBioscience), anti-iNOS (Calbiochem, Merck, Darmstadt, Germany) or anti-cleaved caspase-3 (Cell Signaling, Denver, MA, USA) antibodies. All antibodies were diluted according to manufacturer instructions. Blots were exposed to secondary antibodies: anti-mouse IgG-HRP (Sigma) for tubulin detection, anti-rat-HRP (Nichirei Biosciences Inc.) for CD44 detection and anti-rabbit-HRP (Jackson ImmunoResearch) for iNOS and cleaved caspase-3 detections, followed by enhanced chemiluminescence detection (Biological Industries). Blots were developed using ECL (Molecular imager ChemiDoc XRS+ System, Bio-Rad, CA).

### Glucose clearance and insulin tolerance

Glucose clearance and insulin tolerance tests were assessed by injection of 2g glucose/kg body weight (IPGTT) and injection of 0.75U insulin (Actrapid, Novo Nordisk)/kg body weight (ITT).

### Statistical analysis

Data is presented by means ± SEM and analyzed, unless stated differently, by standard two-tailed Student's t-test equal variance. Histological differences were analyzed by Pearson's χ2 test at P < 0.05, comparing the distribution between the two mouse groups. Generalized Gehan (Breslow) test (Kalbfleisch, J. D.&Prentice, R. L. in The Statistical Analysis of Failure Time Data, Wiley, New York, pp. 16–19,1980) was applied to compare the cumulative incidence of diabetes (survival curve) between the different experimental groups studied. Differences were considered statistically significant when P < 0.05. Statistical analysis was performed using SPSSwin software. Graphs were generated, using either SigmaPlot 10.0 or Excel. Data was processed by Excel.

## Results

### Involvement of CD44 in overt T1D and in reduced motility of islet-infiltrating cells

**Fig 1:**
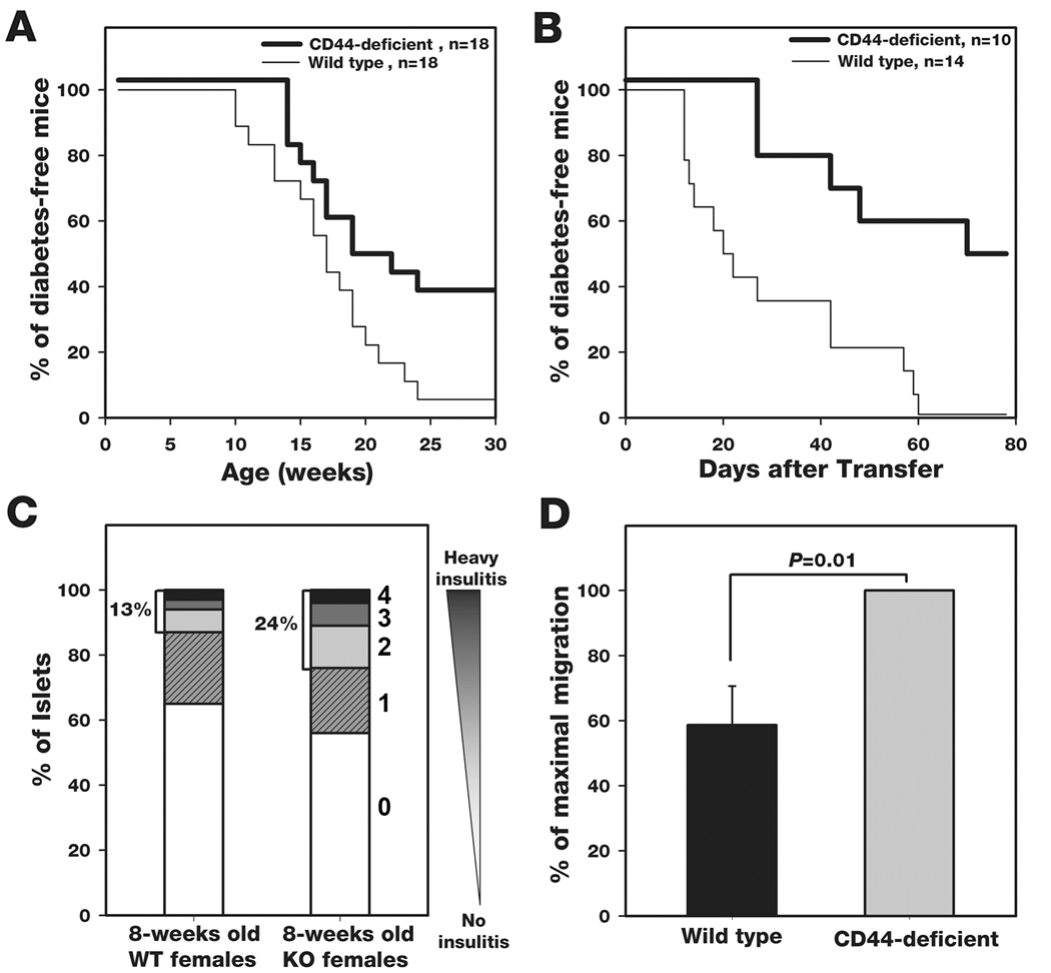
CD44-null NOD females display relative resistance to T1D: role of inflammatory cells. A) and B) Development of T1D in the spontaneous (A) and cell transfer (B) models of WT and CD44-deficient mice was monitored by measuring blood glucose. In the transfer model, irradiated CD44-deficient and WT young (6–8 weeks-old) male recipients were respectively transplanted with splenocytes from CD44-deficient and WT diabetic females. Percentage of diabetes-free mice (showing <250 mg/dL blood glucose) was recorded versus time. Statistical analysis by Breslow; *A*, *P* < 0.05; *B*, *P* < 0.005. C) The invasion capacity of infiltrating cells derived from WT (n = 6) and CD44-deficient (n = 6) NOD females (spontaneous model) was measured as indicated in *Materials and Methods*. A total of 754 pancreatic islets in each mouse group were scored by an uninformed observer. The percentage of islets showing each one of the infiltrating scores was calculated for each mouse, and the average values are presented. Islets from CD44-deficient mice display higher percentage of infiltrating scores (2, 3 and 4) than islets from wild type mice. *P* < 0.0001 by Pearson's χ2 test comparing the distribution of scores in the two mouse groups. D) Wild type or CD44-deficient pre-diabetic spleen cells were added to the top compartment of transwell migration chamber, separated by HA-coated filter (10 μg/filter) from the bottom compartment, and the % of cells migrating through the filter toward the chemoatractant in the bottom compartment was calculated by flow cytometry. Statistical analysis by Student’s-*t-* test. Accumulated data of 4 experiments (n = 8; error bars, SEM).

Our earlier report on the anti-diabetic effect of anti-CD44 mAb or hyaluronidase [13] suggests that interaction between cell surface CD44 and tissue HA enhances the development of T1D in NOD mice. If this is the case, CD44 knockout (KO) females should display a relative resistance to T1D. We generated CD44-deficient NOD mice by backcross breeding of C57BL/6 CD44^-/-^ mice [19] with NOD mice for 12 generations. PCR, Western blot and flow cytometry show an absence of CD44 signals in CD44-null NOD mice (S1A–S1C Fig). As expected, both the spontaneous (Fig 1A) and transfer (Fig 1B) models of NOD mice reveal that CD44-deficient mice are relatively more resistant to T1D than corresponding CD44-positive mice. It should be stressed, however, that in our transfer model of T1D, allo-immunity may be dominant over autoimmunity, because the female donor cells may attack the recipient male islets. Yet, Regardless the mode of inflammation (autoimmune in the spontaneous model and possibly allo-immune in the transfer model), CD44-deficiency induces relative resistance to T1D.

The reduced diabetic activity in CD44-null NOD females could be attributed to an inefficient migratory capacity of CD44-deficient inflammatory cells. However, *ex vivo* histopathological scoring analysis reveals the opposite: inflammatory cells of CD44-deficient NOD females invade the pancreatic islets at higher rates than inflammatory cells of WT mice (Fig 1C), suggesting that the CD44-null cells are more mobile and invasive. In contrast, the WT inflammatory cells tend to be arrested on their substrate and therefore they lose motility [7,9]. Notably, this experiment was performed long before the emergence of diabetes symptoms (8 weeks of age when the mice are normoglycemic) avoiding any bias of group selection. This suggestion is further supported by transwell migration assays performed on total WT and CD44-deficient spleen cells, showing equal distribution of the major cell subsets (CD3^+^, CD4^+^, CD8^+^, B220^+^, CD45^+^). CD44-deficient pre-diabetic splenocytes migrate at higher rates through HA-coated filters than corresponding WT splenocytes (Fig 1D). Although the migration rates of the different cell subsets might be different in the wild type and CD44-deficient groups, we see a correlation between the *in vivo* cell invasion (Fig 1C) and the in vitro cell migration (Fig 1D). Additionally, FACS analyses of spleen, pancreatic lymph nodes, inguinal lymph nodes and islet infiltrates of 8 weeks-old pre-diabetic CD44-deficient and WT NOD females reveal that the CD8/CD4 ratio is similar in both groups (~1:3 in the spleen and lymph nodes, ~1:2 in the pancreatic islets). These findings suggest that the firm binding of inflammatory cells expressing CD44 interferes with their detachment [9] from endothelial HA [10] and slows their migration into the pancreatic islets. Therefore, we envisage that in the absence of CD44 a “spare” molecule (e.g., RHAMM; [16,17,18], possessing weaker adhesion to HA substrate, supports the cell invasion into the pancreatic islet at higher efficacy.

### CD44 expressed in the recipient’s tissue rather than on the infiltrating cells enhances the development of T1D

The major enigma, if not a paradox, of this study is linked to the following question: If WT cells invade pancreatic islets less efficiently than CD44-deficient cells (Fig 1C), why do WT NOD females display less resistance to T1D than CD44-deficient females (Fig 1A and 1B)? To address this puzzle, we hypothesized that, while CD44 positivity attenuates (but does not block) the invasion of inflammatory cells into the pancreatic islets, it also imposes on β cells susceptibility to apoptotic destruction by the infiltrating autoimmune cells. Indeed, CD44 is a wellknown transmitter of apoptotic signals [15].

**Fig 2:**
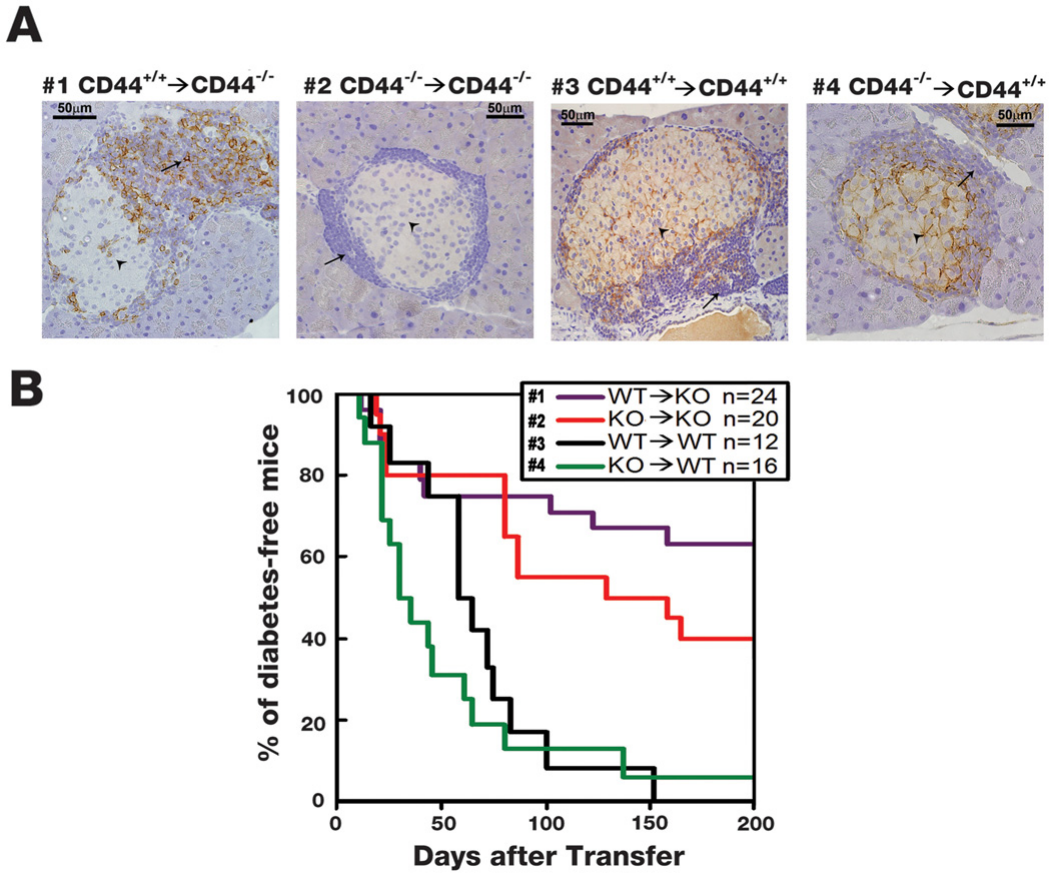
CD44 expressed in the recipient’s tissue rather than on the infiltrating-cells enhances the development of T1D in the cell transfer model. A) CD44-deficient non-diabetic NOD male recipients were reconstituted with inflammatory spleen cells derived from WT (CD44^+/+^→CD44^-/-^; marked #1) or CD44-deficient (CD44^-/-^→CD44^-/-^; marked #2) diabetic NOD female donors. In addition, WT non-diabetic NOD male recipients were reconstituted with inflammatory spleen cells derived from WT (CD44^+/+^→CD44^+/+^; marked #3) or CD44-deficient (CD44^-/-^→CD44^+/+^; marked #4) diabetic NOD female donors. Inflammatory cells are marked by arrows and local islet cells by arrowhead. The pancreases of all 4 combinations were removed after detection of diabetes or 200 days after cell transfer. Doing so, pancreases that were harvested approximately at the same time, were stained with anti-CD44 mAb. B) The % of diabetes-free mice (exhibiting less than 250 mg/dL urine glucose) was determined in corresponding four mouse groups, described in *A*. Statistical analysis by Breslow: #2 versus #3, *P* < 0.01; #1 versus #2 (follow up 75 to 200 days), *P* < 0.03; #3 versus #4 (follow up 20 to 75 days), *P* < 0.03.

To test this prediction we used the cell transfer model of T1D to assess the differential contribution of CD44 expression on infiltrating and islet cells for the development of T1D. Indeed, immunohistochemical analysis, using staining with anti-CD44 mAb, revealed CD44 expression on infiltrating cells, but not in pancreatic islet tissue of CD44-deficient male recipients reconstituted with WT (CD44-positive) diabetic female cells (Fig 2A#1). As expected, CD44 was absent from both infiltrating-cells and islet tissue in CD44-deficient recipients of CD44-deficient diabetic female cells (Fig 2A#2). On the other hand, CD44 was detected in both infiltrating-cells and islet tissue in WT recipients of WT diabetic female cells (Fig 2A#3). Finally, CD44 expression was identified in the islet tissue, but not on infiltrating-cells of WT recipients reconstituted with CD44-deficient diabetic female cells (Fig 2A#4). This immunostaining analysis proves that the cell transfer model allows discrimination between CD44-positive and CD44-negative cells lodging in different islet compartments. Therefore, this model can be used to assess the differential contribution of CD44 expression on infiltrating and islet cells for the development of T1D. Furthermore, the islets represented in Fig 2A were intentionally chosen with moderate infiltration to assess the localization of the CD44 molecule in the different compartments (infiltrate vs. local islet cells). Therefore, and again, the subsets spontaneous versus cell transfer are not comparable.

Using the above model, we found that CD44-deficient NOD recipients of WT or CD44-deficient diabetic donor spleen cells display relative resistance to T1D. In contrast, the WT recipients of WT or CD44-deficient diabetic cells showed enhanced development of the disease (Fig 2B). Furthermore, regardless of whether the recipient mice were CD44-deficient (top lines; #1, 2) or WT (bottom lines; #3, 4), they significantly enhanced the late (75 to 200 days post transfer) phase (top line; #2) or early (20 to 75 days post transfer) phase (bottom line; #4) of T1D when transplanted with CD44-deficient diabetic donor cells (Fig 2B). These findings show that CD44 expression in tissue, rather than on inflammatory cells (i.e., *in vivo* activated spleen cells from diabetic females), dictates the enhancement of T1D. Yet, when the diabetic donor cells are CD44-deficient they reduce, owing to their high mobility, the anti-diabetic effect generated in CD44-deficient NOD recipients and enhance the pro-diabetic effect detected in corresponding WT mice. These results further suggest that CD44-positive WT islet β cells are more susceptible to apoptotic destruction by immune cells than CD44-deficient β cells. Moreover, based on our cell transfer model (Fig 1B), and regardless the method we used to detect glucose in the blood (Fig 1B) or in urine (Fig 2B), we consistently reached the same conclusion: CD44 expression in the tissue rather than in the inflammatory cells confers the pro-diabetic effect.

### Wild type β cells display enhanced susceptibility to apoptosis

**Fig 3:**
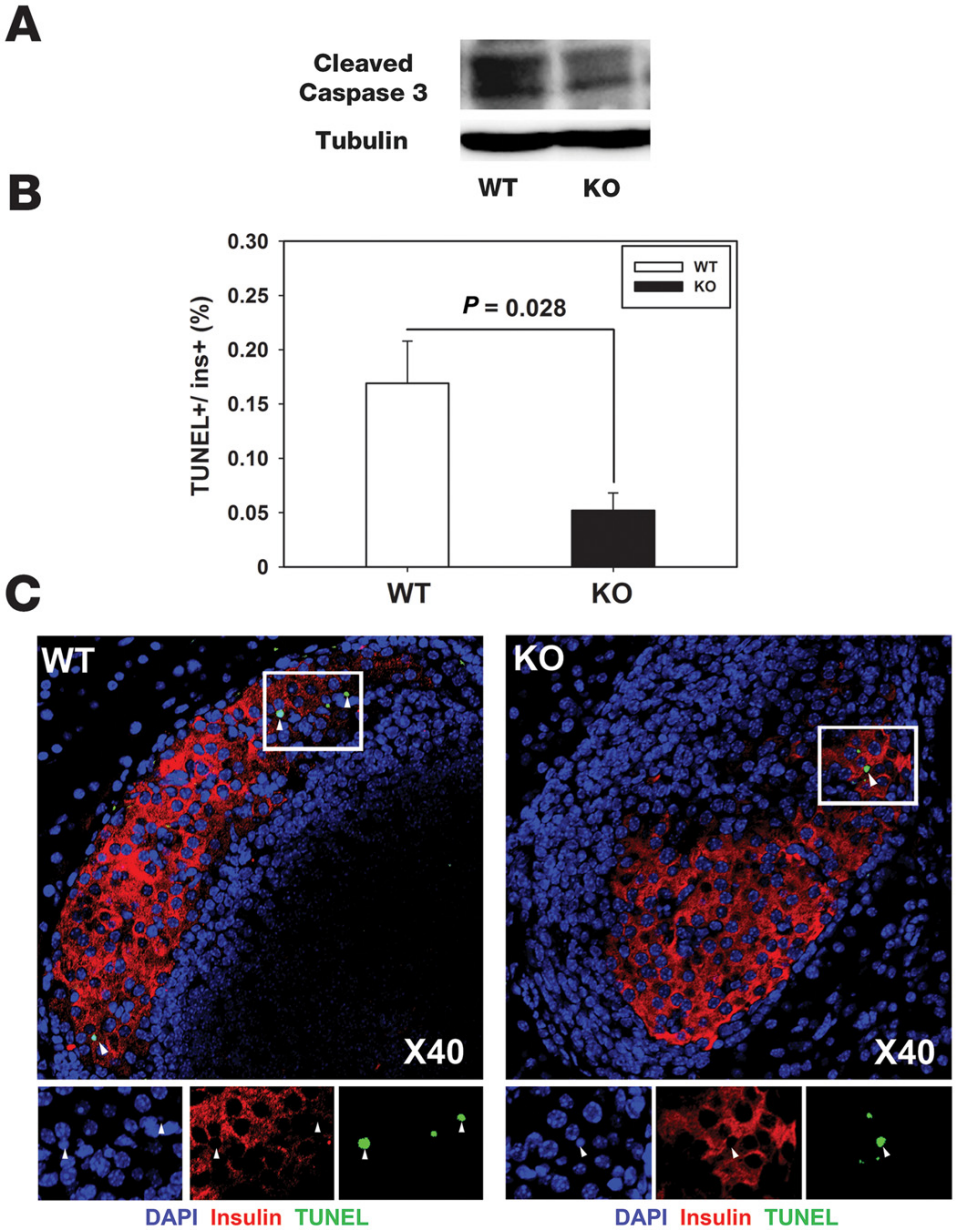
Insulin-producing β cells from WT NOD females display enhanced susceptibility to apoptosis. A) Enhanced caspase-3 activity is detected in cell extracts of WT than in those of CD44-deficient pancreatic islet cells. Pancreatic islet cells were freshly removed from WT and CD44-deficient pre-diabetic NOD females. Cell extracts from islets were subjected to Western blot analysis, using anti-cleaved caspase-3 antibody. A representative Fig of three experiments. (B and C) Pancreata were harvested from 10-weeks-old normoglycemic WT and CD44-deficient NOD females (n = 5 in each group). β-cell apoptosis was assessed using TUNEL assay (green) and co-staining for insulin (red) and DNA (blue). B) Apoptosis was calculated as percent of TUNEL positive nuclei (+/- SEM) in insulin-positive cells out of the total number of insulin-positive cells per mouse. WT (white bar, ~17,560 total β-cells); CD44-deficient (black bar, ~14,800 total β-cells). C) Pictures are representative images of islets from WT (left panel) and CD44-deficient NOD females (right panel). Original images were taken at a magnification of x40. Inset images were digitally increased x5. White arrowheads point to apoptotic β-cells.

CD44-deficient inflammatory cells are more invasive than corresponding WT cells (Fig 1C and 1D). However, CD44-deficient β cells appear to be more resistant to stress and/or apoptosis than corresponding WT β cells, as suggested by Fig 2B. To confirm this suggestion we first showed, that pre-diabetic WT islet β cells express CD44 (S2 Fig), as indicated by double immunofluorescence staining and immunohistochemical staining of rosette forming β cells [22,23]. Pancreatic sections from CD44-deficient NOD mice were stained under identical procedure and the background obtained in these sections was used as reference for CD44 staining. Then we analyzed by Western blot the caspase-3 activity in the cell extracts of pancreatic islets derived from WT and CD44-deficient NOD pre-diabetic females (spontaneous model), and observed an enhanced enzymatic activity in the cell extracts of WT pancreatic islets when compared with those of CD44-deficient (Fig 3A). To substantiate this initial finding, and assure that caspase-3 activity signal is related to insulin-producing β cells, we evaluated the number of apoptotic β cells in the pancreas of prediabetic normoglycemic WT and CD44-deficient NOD females (10-weeks-old). Apoptosis was assessed using the TUNEL assay. Apoptotic cells were calculated as the percent of TUNEL positive nuclei in insulin-positive cells out of the total number of insulin-positive cells per mouse (TUNEL+/ins+). A total of approximately 17,560 and 14,800 β cells were analyzed in the WT and the CD44-deficient groups, respectively. Hence, practically, the number of counted cells is almost the same (*P* = 0.32, N.S.). Fig 3B revealed a 3-fold increase in the percent of WT apoptotic β cells in comparison with the percent of apoptotic β cells calculated in the CD44-deficient group (~0.17%±0.039 apoptotic WT β cells vs. ~0.05%±0.016 apoptotic KO β cells, *P* = 0.028 by Student’s-*t*-test). Fig 3C shows representative images of WT (Fig 3C; left panel) and CD44-deficient (Fig 3C; right panel) pancreatic islets displaying three and one apoptotic β cells, respectively (white arrowheads points toward the relevant cells). It should be taken into account that the vast majority of the apoptotic β cells are quickly phagocytized by cells of the immune system [24]. This result clearly demonstrates that WT β cells are more susceptible to apoptotic killing than corresponding CD44-deficient cells.

**Fig 4:**
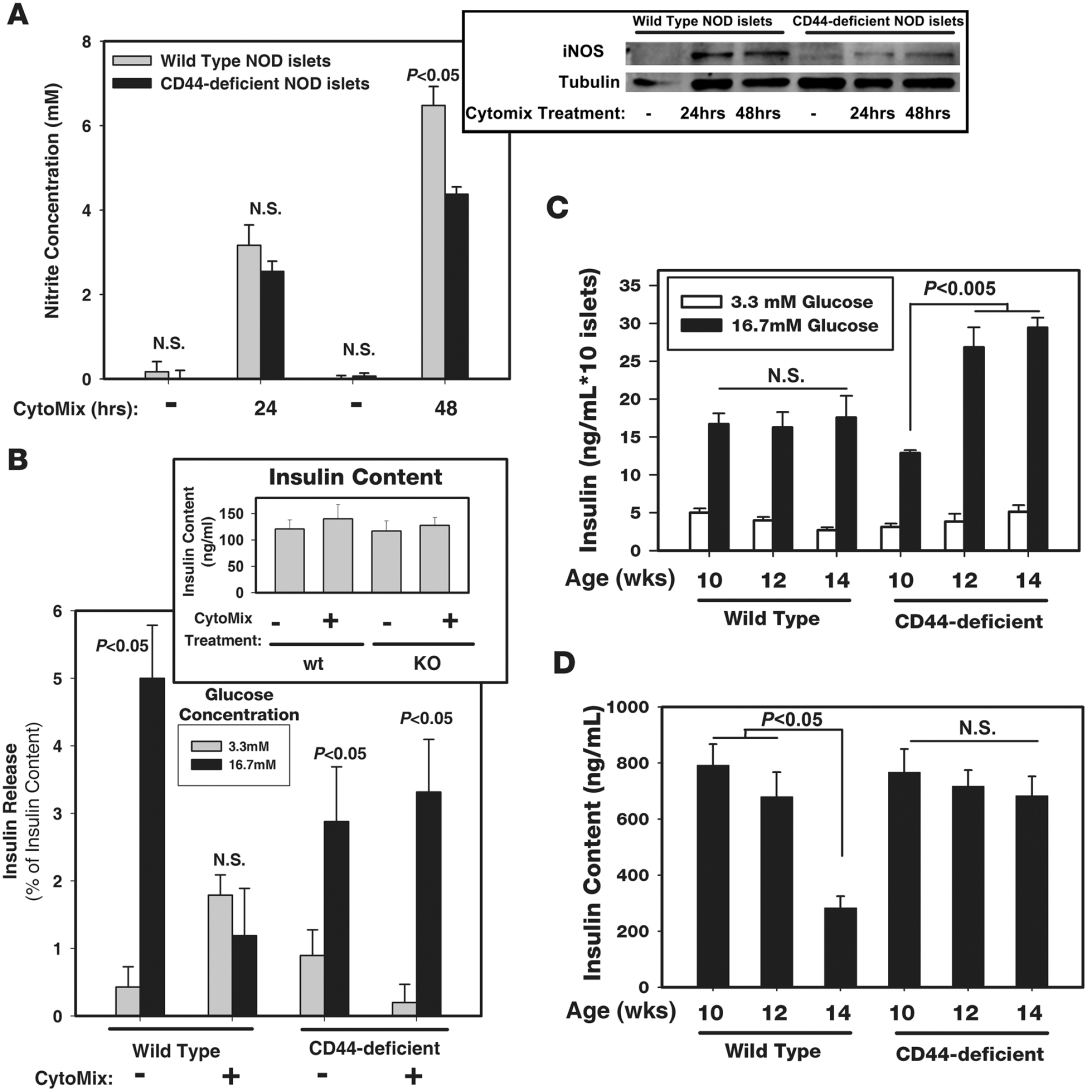
CD44-deficient pancreatic islet cells retain their functionality under pro-inflammatory conditions. (A and B) *In vitro* pro-inflammatory conditions. A) Griess assay. Wild type and CD44-deficient pancreatic islet cells were incubated with medium (-) or Cytomix for 24 and 48 h. Nitrite release was assessed by Griess assay (n = 3). Inset: Cell extracts from pancreatic islets described in (*A*) were subjected to Western blot analysis, using anti-iNOS antibody. A representative Fig of three experiments. B) Glucose-stimulated insulin secretion (GSIS). Pancreatic islet cells from WT and CD44-deficient NOD mice were incubated with medium (-) or Cytomix for 48 h and then stimulated with 3.3 or 16.7 mM glucose for additional one hour and their ability to secrete insulin was measured by ELISA. Inset: Insulin content in each one of the samples (n = 4–5) (error bars, SEM). Statistical analysis for A and B by standard two-tailed Student’s *t*-test. *A*, one representative experiment of five. *B*, one representative experiment of two. (C and D) *Ex vivo* pro-inflammatory conditions. C) Freshly isolated pancreatic islet cells from WT and CD44-deficient pre-diabetic NOD females were processed as described in (*B*) (n = 6–8) (error bars, SEM). D) Content of insulin in pancreatic islets of WT and CD44-deficient pre-diabetic NOD females was analyzed by ELISA. Statistical analysis in *C* and *D* by standard two-tailed Student’s *t*-test. The statistical analysis in *C* shows that the *ex vivo* insulin release of CD44-deficient pancreatic islets at 12 and 14 weeks is significantly higher (p<0.05) than the insulin release of the corresponding WT cells.The statistical analysis in *D* shows that the insulin content in pancreatic islets of 14 wks-old pre-diabetic WT mice is significantly different (*P* < 0.05) from all other samples. (n = 6–8). In *C—D*, at least one representative experiment of two.

Encouraged by these results, we ask whether this reduction in the percent of β cell apoptosis in the CD44-deficient pancreas, is also reflected by an improvement in CD44-deficient β cell functionality when compared with corresponding WT β cells. Nitric oxide (NO), an apoptosis hallmark of β cells, is produced by iNOS in a reaction where arginine and oxygen are converted into citrulline and NO [25]. Therefore, upregulation of iNOS and subsequent NO release are indicators of pancreatic β cell stress. Nitrite accumulation in cell supernatants (indicating NO synthesis) was markedly increased 24 and 48 h after incubation of WT pre-diabetic pancreatic (normoglycemic) islet cells with cytomix, (mixture of the pro-inflammatory cytokines TNF-α, IFN-γ and IL-1β, known to induce apoptosis;[25,26,27,28], when compared with corresponding untreated cells, as shown by Griess reaction [20]. The nitrite release was significantly reduced in CD44-deficient pancreatic islets cells, incubated with the cytokines for 48 h, when compared with corresponding WT cells (Fig 4A). Western blot analysis of iNOS enzymatic activity in the cell extracts of WT and CD44-deficient pancreatic islets further supports this finding (Fig 4A, inset), implying again that CD44-deficient islet cells are more resistant to the *in vitro* cytokine attack than the corresponding WT β cells.

The Glucose-Stimulated Insulin Secretion (GSIS) assay [21] can directly assess damage or impaired function in the islet β cells. Pancreatic islet cells stimulated for 1h with 16.7 mM (but not with 3.3 mM) glucose secrete insulin, as detected by ELISA. Pre-incubation for 48h with cytomix inhibited GSIS in pre-diabetic WT, but not in CD44-deficient islet cells (Fig 4B). Furthermore, the amount of secreted insulin was unchanged after addition of cytomix into CD44-deficient cells (Fig 4B). Note that the insulin content (unlike the insulin secretion) was equal in all cell groups, regardless of cell types (WT or CD44-deficient) and status of cytomix stimulation (Fig 4B, inset; secreted insulin is a small fraction of the insulin content). These results imply that the CD44-null β cells remain more responsive to glucose stimuli under *in vitro* cytokine attack than the WT β cells. This finding further indicates that the expression of CD44 on β cells not only allows (when the cytokines are present) death signaling, but also impairs their function.

To verify whether pancreatic islets from CD44-deficient NOD females retain their responsiveness to glucose stimuli even when facing insulitis, we performed a similar GSIS experiment as described above. However, this time, pancreatic islets were freshly isolated from WT and CD44-deficient pre-diabetic (normoglycemic) NOD females at different ages, without any further stimulation *in vitro*. Fig 4C shows that β cells from 10, 12 and 14 weeks of age of both WT and CD44-deficient mice can be stimulated by the high glucose concentration. Over secretion of insulin by CD44-deficient β cells (*P* < 0.005; Fig 4C) was detected at 12 and 14 weeks of age, possibly to overcome the insulin resistance observed in CD44-deficient NOD females with age (S4C Fig) (this effect is well known in type 2 diabetes) [29]. Note that insulin secretion by CD44-null β cells at 12 and 14 weeks of age was higher (*P* < 0.05) than the corresponding WT cells (Fig 4C). Even more important, we found gradual reduction over time in insulin content of β cells from WT NOD females, whereas such a reduction was not detected in corresponding CD44-deficient females (Fig 4D). This reduced content of insulin in the WT β cells did not affect their responsiveness to high glucose stimulation, presumably because insulin release represents only a small fraction of total β cell content (Fig 4B). This finding shows that β cells from CD44-deficient NOD females facing insulitis retain their insulin content for a longer time than corresponding WT β cells, because they are more resistant to the *in vivo* autoimmune attack.

### Hyaluronic acid—CD44 interaction as a potential trigger of β cell demise

The inflammatory environment induced by the autoimmune process activates hyaluronidase (H’dase), which degrades HA [30]. Hyaluronan of different sizes binds to cell surface CD44 and stimulates apoptotic signals [31,32,33]. Interestingly, a dose of 20U H’dase, injected into WT diabetic cell recipients, restrained the development of T1D (S3A Fig, left panel and [13]). The resistance to T1D, detected in 20U H’dase-treated mice, vanished in CD44-null diabetic cell recipients, which show relative resistance to T1D even in the absence of H'dase (S3A Fig, right panel). This finding suggests that HA binding to CD44 receptor induces apoptosis in β cells and progression of T1D. Indeed, CD44 expression was detected (using both dual-chromogen immunohistochemistry and double immunofluorescence staining) on β cells from WT H’dase-treated mice (S3B Fig upper panels). Immunofluorescence double staining also shows that HA localizes to the membrane of β cells of WT diabetic cell recipients (S3B Fig, bottom panels). In line with S3B Fig (HA and CD44 localizations on β cells membrane), Western blot demonstrates that HA binding to islet cells upregulates cleaved caspase-3 in islets of WT DBA/1 mice, but not in islets of corresponding CD44-deficient mice (S3C Fig), suggesting that CD44-null islet cells relatively resist apoptotic death induced by HA. β cells from WT DBA/1 mice, rather than from NOD mice, were analyzed to avoid “noise” that may be generated by inflammatory cells invading the NOD mouse islets, but not the DBA/1 mouse islets. Indeed, cytokine–confronted β cells from mouse (e.g., [34]) and rat (e.g., [35]) are frequently used to study apoptosis in these cells. These findings suggest that HA binding to CD44 receptor induces apoptosis in β cells and progression of T1D.

### CD44-dependent glucose uptake by peripheral cells

Unexpectedly the intra-peritoneal glucose tolerance test (IPGTT) showed that WT NOD females, cleared blood glucose (following glucose injection) faster than the corresponding CD44-deficient females (S4A and S4B Fig). We therefore suggest that the insulin-dependent glucose uptake by peripheral cells is also a CD44-dependent event. Indeed, the intra-peritoneal insulin tolerance test (ITT) revealed that CD44-deficient NOD females, as well as CD44-deficient non-diabetic DBA/1 mice, exhibit decreased insulin sensitivity following insulin injection than corresponding WT females (S4C and S4D Fig).

## Discussion

**Fig 5:**
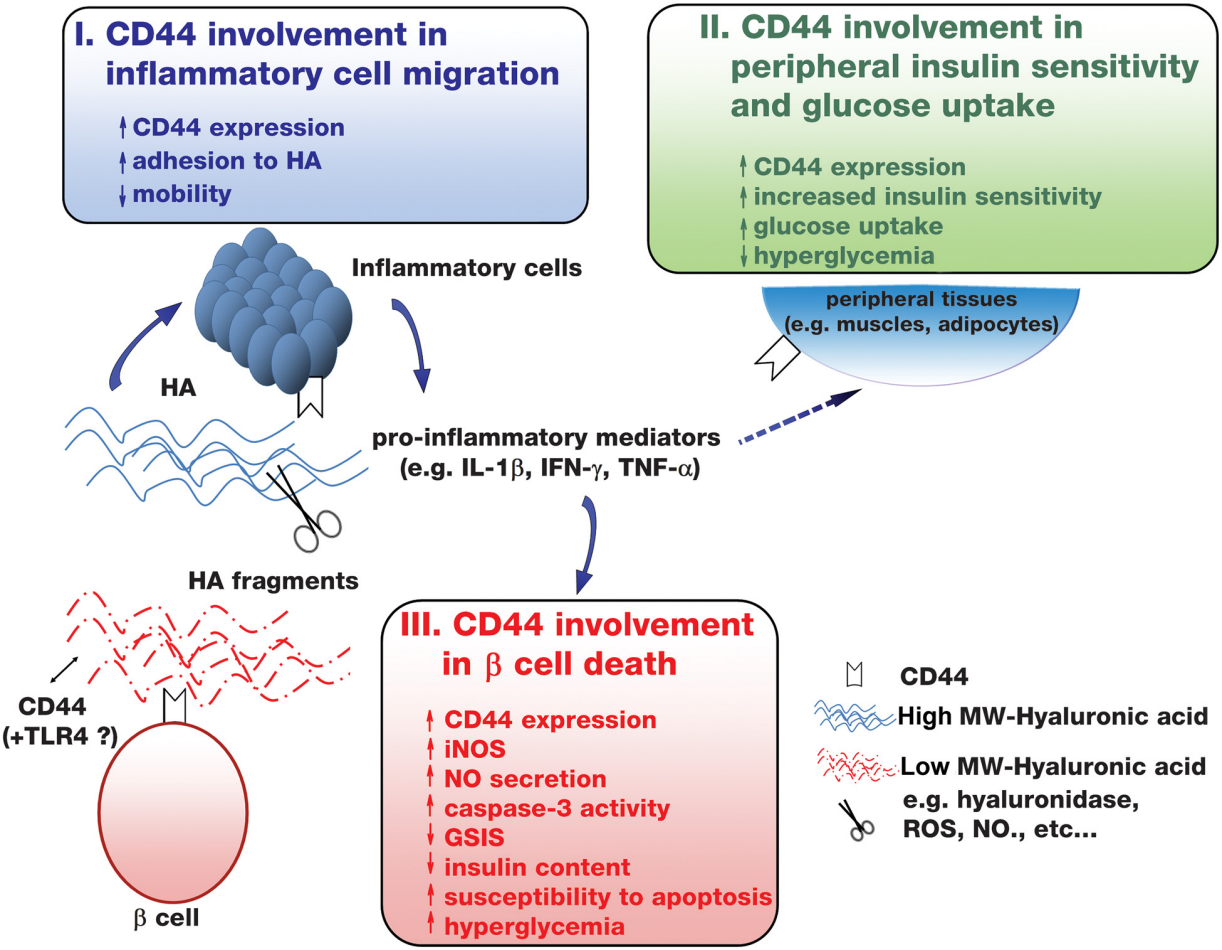
CD44 involvement in T1D. Three CD44-dependent pathways affect the fate of T1D in NOD mice. I. CD44 involvement in inflammatory cell migration (blue). Inflammation upregulates CD44 on the islet-infiltrating cells. The firm adhesion of the CD44 receptor to the HA substrate slows the motility of the inflammatory cells on the endothelium. II. CD44 involvement in peripheral insulin sensitivity and glucose uptake (green). CD44 expression on peripheral tissue, increases insulin sensitivity and glucose uptake by, for example, muscle and liver cells, resulting in reduced hyperglycemia. Compensation for insulin-deficiency (see pathway III) may explain this event. III. CD44 involvement in β cell apoptotic death (red). Inflammation-induced cytokines up-regulate CD44 expression on β cells. CD44 expression on β cells is associated with increased β cell dysfunction and susceptibility to apoptosis, which could be triggered by the binding of HA fragments to CD44 receptor. However, large HA fragments could interfere with the detrimental effect of LMW-HA on β cells. The β cell dysfunction and susceptibility to apoptosis is indicated by increased iNOS induction and subsequent NO production, increased caspase-3 activity (Western blot, not shown), reduced glucose-stimulated insulin secretion, and reduced insulin content. As an outcome of β cell demise, hyperglycemia is detected, implying the development of T1D. Broken arrows and question marks represent pathways and factors that are yet to be established.

The triple equilibrium which determines the fate of T1D in NOD mice is illustrated in Fig 5. The relative protective effect of CD44 expression on inflammatory cells, which attenuates insulitis possibly through reduced cell motility, and its expression on peripheral cells, which increases insulin sensitivity, did not prevent the progression toward overt diabetes, suggesting that CD44 expression on β cells could be detrimental. Additionally, it should be indicated that insulin secretion may be a CD44-dependent event since CD44-deficient β cells secrete basically (in the absence of cytomix/inflammation) less insulin than corresponding WT cells (Fig 4B). Therefore, it is possible that CD44 also has a supportive effect on insulin release from β cells. A deepen analysis of the involvement of CD44 in insulin secretion is a matter of further investigation.

The suggested integration of the above described CD44-related factors (Fig 5) with already known mechanisms associated with T1D development may deepen our understanding of key factors leading to disease progression. In addition, these findings may be important to understand other inflammatory and malignant diseases. To this end, it has been reported that expression of CD44 on cancer cells impose on them apoptotic susceptibility, which can be exploited for therapeutic targeting [36]. Also, over expression of CD44 in tissues of the central nervous system may confer on them susceptibility to apoptotic killing leading to neurodegenerative diseases.

### Involvement of CD44 in reduced mobilization of inflammatory cells

We [7] and Savinov et al. [9] have reported that CD44 expressed on hematopoietic progenitor cells and inflammatory diabetogenic cells can arrest these cells in tissues (bone marrow and endothelium, respectively). In addition, CD44 cleavage by MT1-MMP allows their release and motility in the vascular system [7,9]. It further appears that inflammatory cells expressing CD44 are, in contrast to CD44-deficient cells, partially arrested on HA-coated filters and their invasion into pancreatic islet is reduced. These findings are in line with the notion that CD44 expression on inflammatory cells reduces their motility, but does not prevent their infiltration into the pancreatic islets, consistent with our earlier report [13]. The enhanced invasion of CD44-null inflammatory cells could be related to a relatively weak attachment of their cell surface molecule, which redundantly replaces CD44 in CD44 knockout mice [16], to the same HA ligand. Based on our cell migration studies (Fig 1) and previous communications [7,9,16], we suggest that cells that do not express CD44 but rely only on a redundant cell surface molecule are more motile and invasive than cells expressing CD44. In agreement, neutrophil accumulation in the lungs of CD44-deficient mice infected with *E*. *coli*-induced pneumonia is more intense than in lungs of corresponding WT mice [37].

Our on-going experiments suggest that, as in collagen-induced arthritis (CIA) [16], RHAMM [17,18] is the redundant molecule that compensates for CD44 when CD44 is genetically deleted. While evidence for compensation by RHAMM in CD44- deficient mice is still a matter for further investigation, we suggest that—the redundant molecule possesses weaker adhesion to HA substrate than CD44 and therefore shows higher efficacy in support of cell invasion into arthritic joints [16] and possibly into pre-diabetic pancreatic islets.

### Involvement of CD44 in apoptotic death of β cells

CD44 is a well-known transmitter of apoptotic signals [15,38]. We report here that *in vitro* incubation of WT (CD44-positive) pancreatic islet cells with HA or with a cocktail of pro-inflammatory cytokines results in β-cell damage or dysfunction, which could be a consequence of upregulation of apoptotic signals (e.g., caspase-3) or enhanced release of NO and insensitivity of β cells to glucose stimulation (S3 Fig and Fig 4). These biomarkers were decreased or were absent in CD44-deficient mice. Unfortunately, owing to built-in staining limitation, we were unsuccessful to provide evidence of dual staining of both CD44 and HA on insulin-positive cells. Although this limitation must be taken into a consideration, combining the data provided in S2 and S3A Figs, suggests that this event may occur either by a direct binding of HA to β cell CD44 or by binding of HA to β cell Toll-like receptor-4 (TLR-4) [39], which is included in TLR-4/CD44 signaling complex [40]. In addition, our conclusion must be further reserved by the fact that CD44 is expressed not only on β cells, but also on many other cells in the diabetic pancreas. The elucidation of the entire signaling pathway generated after such a binding, is a matter for further investigation. The *in vitro* studies were confirmed by corresponding *ex vivo* experiments using pancreatic β-cells taken from pre-diabetic normoglycemic NOD females (e.g., see the TUNEL assay). These findings imply that WT β cells may display a potential apoptotic susceptibility mediated by CD44 and that CD44-deficient β cells are relatively resistant to inflammation-mediated damage. Here again, our conclusion are based mostly on the functional studies, while the direct detrimental effect of HA binding to β cell CD44 is circumstantial.

### Involvement of CD44 in insulin-dependent glucose uptake

The observation that blood glucose clearance was reduced in CD44-deficient mice, whereas CD44-deficient mice display relative resistance to T1D, encouraged us to ask whether glucose uptake by peripheral cells and β cells [41,42] is a CD44-dependent event, as it has been shown for cancer cells [43]. Indeed we found (S4 Fig) that WT NOD females clear blood glucose faster than the corresponding CD44-deficient females in response to an insulin injection. This finding suggests that CD44-expressing cells present in the periphery, display stronger sensitivity to insulin-dependent glucose uptake than the corresponding CD44-deficient cells. Along this line, two reports have recently implicated the CD44 receptor in adipose tissue inflammation and type 2 diabetes [44,45].

The arguments supporting the finding that CD44 expression renders β cells susceptible to inflammatory attack (Fig 3) are based on functional assays restricted to β cells such as GSIS (Fig 4B and 4C) and determination of insulin content (Fig 4D). The use of WT and CD44-null NOD mice allowed us to unveil the role of CD44 in inflammatory cells, peripheral cells and β cells. Had we worked on NOD mice with inducible CD44-knockout β cells, we would not have been able to unveil these effects. Naturally, CD44 is not the only factor that dictates the development of the disease, and the weight of the CD44 contribution to the pathology could be different in different individuals. For example, the concept that CD44-deficient β cells are relatively resistant to apoptosis is based on the average of the total mouse population. Some individual CD44-null mice could be as susceptible as WT mice to the disease development owing to the weight of the other factors (e.g., influence of CD44 on Treg activity in T1D). The situation is even more complicated in an outbred population, where the extreme ends studied here (wild type vs. CD44-deficiency) do not exist. However, even for an outbred population, our findings suggest that the differential effects of CD44 on inflammatory cells, peripheral cells or β cells, together with the contribution of other factors (e.g., the individual’s genetic profile) determine whether the balance is shifted towards disease enhancement or restraint. This conclusion is valid for other inflammatory and cancer diseases in which CD44 has a bioactive role.

## Supporting Information

S1 Fig Analysis of CD44 expression in Wild type (WT) and CD44-deficient NOD mice. A) PCR analysis of tail DNA discriminates between WT (#1, 120bp product band), CD44-deficient (#2, 300bp product band) and heterozygous (#3, both bands) mice. (-) PCR negative control including all the PCR compounds except DNA. B) Western blot analysis of spleen cell extract subjected to 10% SDS-PAGE followed by immunoblotting with anti-CD44 antibody and C) flow cytometry analysis with 0.5 μg/ml IM7.8.1 FITC-conjugated anti-CD44 mAb (eBioscience, see [16]) reveal CD44 signal in WT, but not in CD44-deficient splenocytes. IgG2b, Isotype-matched control antibody.
(TIF)

S2 Fig Insulin-secreting β cells express CD44. A) Immunofluorescence double staining of pancreatic islets from WT recipients of WT diabetic cells (WT>WT) with second antibody alone (top left panel) as well as with anti-insulin (top right panel; green) and anti-CD44 (bottom left panel; red) antibodies. DAPI (blue) was used to stain the nuclei. The red dots observed in the top left panel are probably background staining of erythrocytes. Background from CD44-knockout pancreatic sections was subtracted in all analyses. The merge of the two antibodies yielded a yellow color (bottom right panel). B) Immunohistochemistry staining of CD44-positive rosette-forming cells (marked by arrowheads; magnification x 2 in inset), which are typically characterize β cells [22,23]. Scale bars indicate the magnification size.
(TIF)

S3 Fig CD44-HA interaction enhances T1D by inducing islet apoptosis. A) Cell transfer assay. Wild type (left panel) and CD44-null (right panel) irradiated NOD male recipients were respectively transplanted with splenocytes from WT and CD44-deficient diabetic NOD females. One hour before cell transfer and then, every other day, the mice were subjected to injections (three injections/week for 4 weeks, a total of 12 injections) of either PBS or hyaluronidase (H’dase) (20U). Percent of diabetic cell recipients free of diabetes was recorded versus days after cell transfer. Statistical analysis by Breslow. B) CD44 and HA localization on β cells. Double immunofluorescence (upper left and bottom panels) and dual-chromogen staining (upper right panel). Sections (top and both bottom panels) from pancreatic islets derived from H’dase-treated WT cell recipients were subjected to double fluorescence staining with anti-insulin (green) and anti-CD44 (5 μg/ml; red) or biotinylated HABP (2.5 μg/ml; red), as described. DAPI staining was used to detect cell nuclei. Sections analyzed by confocal microscopy revealed that CD44 (upper left panel, red) and HA (bottom panels, red) are localized on β cell membrane (green). Immunohistochemistry with two chromogens confirms the presence of CD44 on insulin-positive β cells (upper right panel, dark grey, CD44; red, insulin). C) Western blot. Islet cells from WT and CD44-deficient DBA/1 mice were incubated for 48h with 300 μg/ml HA and then subjected to Western blot analysis, using anti-caspase-3 antibodies. One representative experiment of two.
(TIFF)

S4 Fig CD44-dependent glucose uptake by peripheral cells. (A and B) Intra-peritoneal glucose tolerance test: CD44-deficient NOD females show impaired glucose clearance. Overnight-fasted normoglycemic WT (black circles) and CD44-deficient (white circles) NOD females (n = 7 mice in each group) of the indicated ages were i.p. injected with glucose (2 gr/kg) and the clearance of glucose from the blood was measured by blood glucose determination (mg/dL) 0, 15, 30, 60 and 120 min after the first glucose injection. The glucose clearance at 11 weeks of age is shown in A and the area under the curve (AUC) analysis at different mouse ages, is shown in B (WT- black bars; CD44-deficient- grey bars). AUC is the trapezoidal rule, which determines the area under the curve, using Excel software. Data presented are means ± SEM. (C and D) Intra-peritoneal insulin tolerance test: CD44-deficient NOD females show decreased insulin sensitivity. Overnight-fasted WT (black circles) and CD44-deficient (white circles) NOD females (n = 5–6 mice in each group), 14 weeks of age (C), as well as normal DBA/1 mice 8 weeks of age (D), were i.p. injected with insulin (0.75 units/kg; Actrapid, Novo Nordisk, Denmark) and the clearance of glucose from the blood was measured by determination of percent of blood glucose at 0, 20, 40, 60 and 80 min after the insulin injection. Blood glucose concentration (mg/dL) at time 0: NOD mice, WT: 68.3±2.8; CD44-null: 57.2±2.3. DBA/1 mice, WT: 70.8±1.6; CD44-null: 74.2±2.4. In A—D, Statistical analysis by 2-tailed invariant Student’s t-test. * P < 0.05; ** P < 0.005.
(TIF)
