# Supplementary figures and images for: Can CD44 Be a Mediator of Cell Destruction? The Challenge of Type 1 Diabetes
Source: PLoS One. 2015 Dec 1;10(12):e0143589. doi: 10.1371/journal.pone.0143589 (PMC4666674; doi:10.1371/journal.pone.0143589)

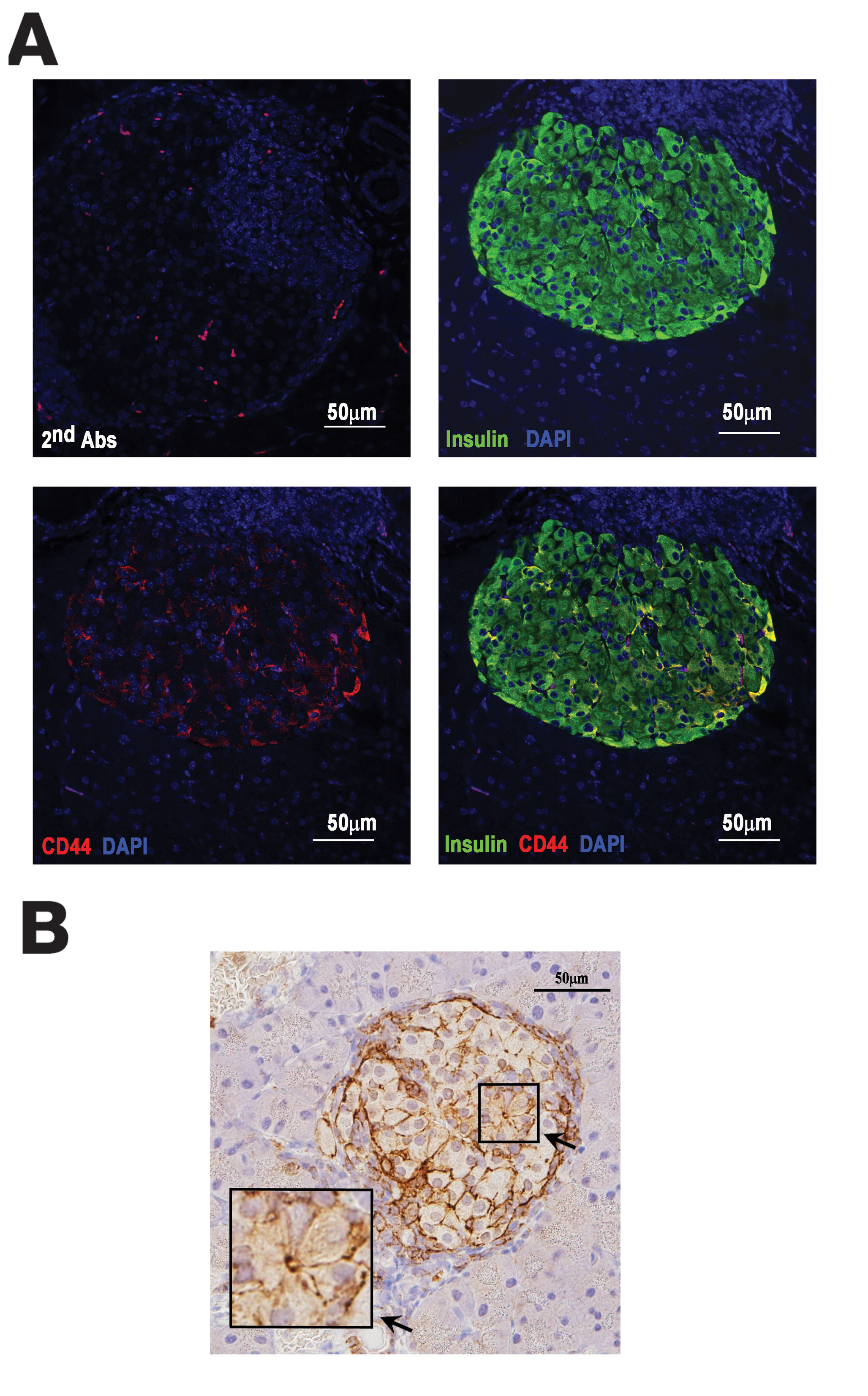

Supplement: S2 Fig — A) Immunofluorescence double staining of pancreatic islets from WT recipients of WT diabetic cells (WT>WT) with second antibody alone (top left panel) as well as with anti-insulin (top right panel; green) and anti-CD44 (bottom left panel; red) antibodies. DAPI (blue) was used to stain the nuclei. The red dots observed in the top left panel are probably background staining of erythrocytes. Background from CD44-knockout pancreatic sections was subtracted in all analyses. The merge of the two antibodies yielded a yellow color (bottom right panel). B) Immunohistochemistry staining of CD44-positive rosette-forming cells (marked by arrowheads; magnification x 2 in inset), which are typically characterize β cells [22,23]. Scale bars indicate the magnification size. (TIF) [file pone.0143589.s002.tif]

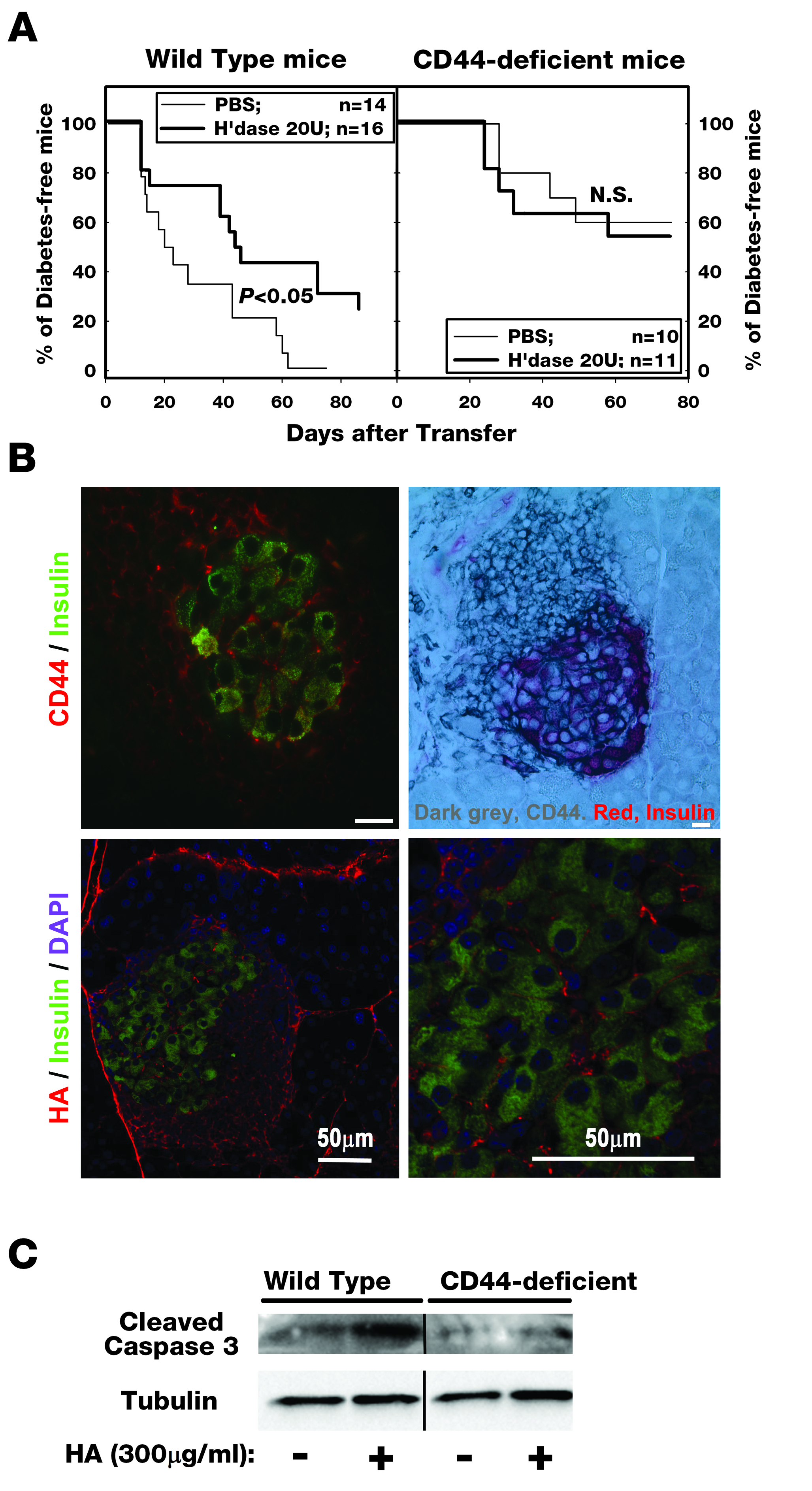

Supplement: S3 Fig — A) Cell transfer assay. Wild type (left panel) and CD44-null (right panel) irradiated NOD male recipients were respectively transplanted with splenocytes from WT and CD44-deficient diabetic NOD females. One hour before cell transfer and then, every other day, the mice were subjected to injections (three injections/week for 4 weeks, a total of 12 injections) of either PBS or hyaluronidase (H’dase) (20U). Percent of diabetic cell recipients free of diabetes was recorded versus days after cell transfer. Statistical analysis by Breslow. B) CD44 and HA localization on β cells. Double immunofluorescence (upper left and bottom panels) and dual-chromogen staining (upper right panel). Sections (top and both bottom panels) from pancreatic islets derived from H’dase-treated WT cell recipients were subjected to double fluorescence staining with anti-insulin (green) and anti-CD44 (5 μg/ml; red) or biotinylated HABP (2.5 μg/ml; red), as described. DAPI staining was used to detect cell nuclei. Sections analyzed by confocal microscopy revealed that CD44 (upper left panel, red) and HA (bottom panels, red) are localized on β cell membrane (green). Immunohistochemistry with two chromogens confirms the presence of CD44 on insulin-positive β cells (upper right panel, dark grey, CD44; red, insulin). C) Western blot. Islet cells from WT and CD44-deficient DBA/1 mice were incubated for 48h with 300 μg/ml HA and then subjected to Western blot analysis, using anti-caspase-3 antibodies. One representative experiment of two. (TIFF) [file pone.0143589.s003.tiff]

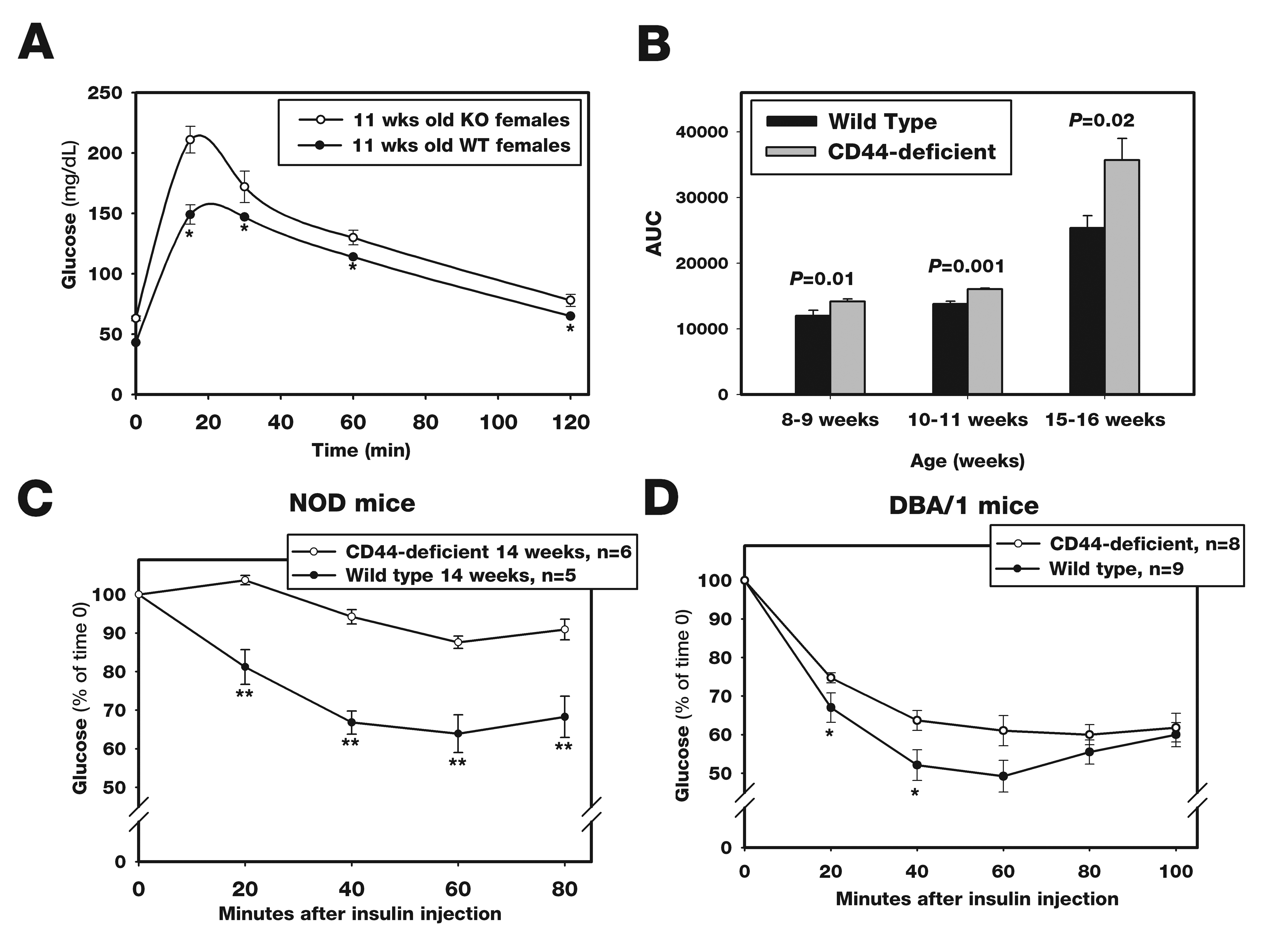

Supplement: S4 Fig — (A and B) Intra-peritoneal glucose tolerance test: CD44-deficient NOD females show impaired glucose clearance. Overnight-fasted normoglycemic WT (black circles) and CD44-deficient (white circles) NOD females (n = 7 mice in each group) of the indicated ages were i.p. injected with glucose (2 gr/kg) and the clearance of glucose from the blood was measured by blood glucose determination (mg/dL) 0, 15, 30, 60 and 120 min after the first glucose injection. The glucose clearance at 11 weeks of age is shown in A and the area under the curve (AUC) analysis at different mouse ages, is shown in B (WT- black bars; CD44-deficient- grey bars). AUC is the trapezoidal rule, which determines the area under the curve, using Excel software. Data presented are means ± SEM. (C and D) Intra-peritoneal insulin tolerance test: CD44-deficient NOD females show decreased insulin sensitivity. Overnight-fasted WT (black circles) and CD44-deficient (white circles) NOD females (n = 5–6 mice in each group), 14 weeks of age (C), as well as normal DBA/1 mice 8 weeks of age (D), were i.p. injected with insulin (0.75 units/kg; Actrapid, Novo Nordisk, Denmark) and the clearance of glucose from the blood was measured by determination of percent of blood glucose at 0, 20, 40, 60 and 80 min after the insulin injection. Blood glucose concentration (mg/dL) at time 0: NOD mice, WT: 68.3±2.8; CD44-null: 57.2±2.3. DBA/1 mice, WT: 70.8±1.6; CD44-null: 74.2±2.4. In A—D, Statistical analysis by 2-tailed invariant Student’s t-test. * P < 0.05; ** P < 0.005. (TIF) [file pone.0143589.s004.tif]
